# El Niño and other climatic drivers of epidemic malaria in Ethiopia: new tools for national health adaptation plans

**DOI:** 10.1186/s12936-023-04621-3

**Published:** 2023-06-24

**Authors:** Adugna Woyessa, Asher Siebert, Aisha Owusu, Rémi Cousin, Tufa Dinku, Madeleine C. Thomson

**Affiliations:** 1 Ethiopian Public Health Institute, P.O. Box 1242/5654, Addis Ababa, Ethiopia; 2 International Research Institute for Climate and Society, Columbia University, PO Box 1000, Palisades, NY 10964 USA; 3 College of Atmospheric and Geographical Sciences, Oklahoma University, Norman, OK USA

**Keywords:** Climate variability and change, Adaptation, Ethiopia, Rainfall temperature ENSO, ENACTS, Malaria, Epidemic

## Abstract

**Background:**

Ethiopia has a history of climate related malaria epidemics. An improved understanding of malaria–climate interactions is needed to inform malaria control and national adaptation plans.

**Methods:**

Malaria–climate associations in Ethiopia were assessed using (a) monthly climate data (1981–2016) from the Ethiopian National Meteorological Agency (NMA), (b) sea surface temperatures (SSTs) from the eastern Pacific, Indian Ocean and Tropical Atlantic and (c) historical malaria epidemic information obtained from the literature. Data analysed spanned 1950–2016. Individual analyses were undertaken over relevant time periods. The impact of the El Niño Southern Oscillation (ENSO) on seasonal and spatial patterns of rainfall and minimum temperature (Tmin) and maximum temperature (Tmax) was explored using NMA online Maprooms. The relationship of historic malaria epidemics (local or widespread) and concurrent ENSO phases (El Niño, Neutral, La Niña) and climate conditions (including drought) was explored in various ways. The relationships between SSTs (ENSO, Indian Ocean Dipole and Tropical Atlantic), rainfall, Tmin, Tmax and malaria epidemics in Amhara region were also explored.

**Results:**

El Niño events are strongly related to higher Tmax across the country, drought in north-west Ethiopia during the July–August–September (JAS) rainy season and unusually heavy rain in the semi-arid south-east during the October–November–December (OND) season. La Niña conditions approximate the reverse. At the national level malaria epidemics mostly occur following the JAS rainy season and widespread epidemics are commonly associated with El Niño events when Tmax is high, and drought is common. In the Amhara region, malaria epidemics were not associated with ENSO, but with warm Tropical Atlantic SSTs and higher rainfall.

**Conclusion:**

Malaria–climate relationships in Ethiopia are complex, unravelling them requires good climate and malaria data (as well as data on potential confounders) and an understanding of the regional and local climate system. The development of climate informed early warning systems must, therefore, target a specific region and season when predictability is high and where the climate drivers of malaria are sufficiently well understood. An El Niño event is likely in the coming years. Warming temperatures, political instability in some regions, and declining investments from international donors, implies an increasing risk of climate-related malaria epidemics.

**Supplementary Information:**

The online version contains supplementary material available at 10.1186/s12936-023-04621-3.

## Background

### Malaria and climate in Africa

Malaria is a climate-sensitive disease transmitted by *Anopheles* mosquitoes. In Africa, three species, namely *Anopheles gambiae, Anopheles arabiensis* and *Anopheles funestus,* are responsible for most malaria transmission. The life cycle of these mosquitoes, and the malaria parasites (*Plasmodium* spp.) they carry, are highly sensitive to climatic and environmental factors with temperature being a significant driver of the development rates of both the mosquito vector and the malaria parasite. In addition, rainfall, stagnant water bodies and humidity provide essential environmental conditions for juvenile mosquito development and adult survivorship [1]. Because there are lags between the climate drivers and the development of epidemics there is the potential to develop climate-based early warning systems [2]. Health early warning systems are currently being considered as a component of national adaptation plans [3].

Africa has a long history of climate-related malaria epidemics [4]. These have declined in recent years as a result of the extensive control measures instigated since the turn of the twenty-first century. However, despite significant national and international investments, the continent continues to bear the brunt of global malaria deaths and cases [5]. A funding hiatus in recent years and the COVID-19 pandemic has significantly undermined control efforts putting the continent at risk of malaria resurgence [6]. Combined with political strife, cyclical climate drivers, such as the El Niño Southern Oscillation (ENSO), and global warming, there is a real risk that malaria epidemics will re-emerge on the continent if renewed political will is not forthcoming. There are new opportunities to use climate information in malaria control and elimination (as well as other climate sensitive health risks) because of greater collaboration between the public health and climate communities [7].

Understanding the spatial and temporal relationships of climate drivers to malaria transmission is important in order to predict epidemics [8], better understand the attributable impact of malaria interventions [9], and climate proof malaria eradication strategies in a warming world [10].

The seasonal, year to year and longer-term variations in the climate (rainfall, temperature) that drive vector-borne diseases in Africa are substantially driven by slowly evolving oceanic processes, particularly the sea surface temperatures (SSTs) in the Pacific [11], Indian [12], and Tropical Atlantic [13] oceans. Geographic regions and seasons that are strongly impacted by SSTs have an increased likelihood of skilful seasonal climate forecasts, which may be used to increase the lead time for climate-based malaria early warning systems [14, 15].

ENSO is the most important driver of climate variability around the world at seasonal-to-interannual timescales and a major source of climate predictability, especially in the tropics [16]. El Niño (unusually warm SSTs) and La Niña (unusually cool SSTs) in the eastern pacific are the positive and negative phases of the ENSO cycle; between these two phases is a third phase; ENSO-neutral. Unlike the rapidly changing atmosphere which drives the weather, oceanic conditions may persist for months. El Niño and La Niña events typically persist for 9–12 months or longer, developing in the northern summer and typically peaking between November and January. The impact of ENSO on a range of climate sensitive health outcomes (including malaria, dengue, and diarrhoeal disease) has been documented [17]. However, the relationships of ENSO to climate and associated health impacts are complex as noted in the publication “Climate drivers of infectious diseases in Africa” [8]. The authors found that ENSO (El Niño and La Niña) impact the climate (and thereby climate-sensitive health outcomes): (a) differently according to the variable of interest (e.g. rainfall, and minimum and maximum temperature), (b) at different spatial scales, (c) in some regions and not others, (d) in some seasons and not others, (e) often according to its strength, and sometimes in a non-linear fashion, (f) at varying periods (from 5 months to 2 years), with both El Niño and La Niña events on occasions occurring in the same calendar year (e.g., 2010), (g) often substantially conditioned on the action of other oceanic climate drivers.

The rainfall response to ENSO is often nearly contemporaneous, with related rainfall impacts generally occurring during a region’s traditional rainy season [18]. Short term (1–2 years) increases in temperatures occur across the tropics during and immediately following an El Niño event. The local impact of El Niño on temperature will be moderated by the rainfall response to ENSO as cloud cover will reduce day-time temperatures but lead to warmer nights [19]. The Indian Ocean Dipole (IOD) is, in many ways, analogous to ENSO but impacts on rainfall at a regional level (e.g., Eastern/Southern Africa) rather than at the global level. SSTs in the Gulf of Guinea (the Tropical Atlantic) have been shown to influence rainfall in the Sahel and that influence can extend to western Ethiopia [13].

To date, concomitant changes in climate and malaria transmission in the highlands of Eastern Africa have been noted at seasonal [20], interannual [21], decadal [22], and multi-decadal [23], timescales. In Southern Africa, seasonal climate forecasts, influenced by SSTs have been shown to be predictive of annual changes in national malaria cases once long-term trends have been removed [15]. However, such forecasts are invariably large scale [14]. Subnational analysis, suitable for local, operational decision-making, is dependent on high quality local climate observations, which can be used to better assess climate and malaria relationships as well as downscale seasonal forecasts at local levels.

### The climate of Ethiopia

The Ethiopian climate is extremely complex because of its varied topography and geographic location. Situated on the Horn of Africa it bridges the climate systems of Eastern Africa and the Sahel. Consequently, it has three distinct rainy seasons [24]. In central and eastern Ethiopia these are: (1) the dry (October to January: ONDJ), (2) the short rainy (February to May FMAM), and (3) the main rainy (June to September: JJAS) seasons. These seasons are locally defined as *Bega*, *Belg* and *Kiremt*, respectively. In south-east and southern locations, the first two seasons correspond with the main East African seasons of October–November-December (OND) and March–April–May (MAM) whereas the third season, June–July–August (JJA) is mostly dry. However, for much of central and northern Ethiopia, the main rainy season is the JJAS *Kiremt* season, which corresponds with the timing of the Sahelien rainy season. A precise delineation of distinct climate regions and rainy seasons is difficult, as Ethiopia’s climate can vary substantially within a short distance [25].

In Ethiopia, El Niño is associated with drought in the north-west region during the *Kiremt* season [26, 27], and increasing rainfall in the south during the short *Belg* season. La Niña is associated with approximately opposite conditions, with higher than average rainfall in the *Kiremt* season in the north-west region [28]. More recent forecasting studies have shown that the variability of the *Kiremt* season can be fairly well reproduced for much of northern and north-eastern Ethiopia using the North American Multi-Model Ensemble (NMME) [29], and that the coupling between ENSO and Ethiopian *Kiremt* rainfall is reproduced effectively by the European Centre for Medium Range Weather Forecasting (ECMWF-SEAS5) model [30]. The Indian Ocean Dipole has an important influence on the climate of East Africa. Both the Indian and Tropical Atlantic oceans are important moisture sources for Ethiopian rainfall. Furthermore, evidence suggests that extreme droughts in Ethiopia will likely increase in association with climate change [31].

Short-term warming has been observed in association with El Niño events and poses a significant risk for malaria transmission in Ethiopia [32]. Simple linear regression and spatial analyses have been used to associate SSTs in the Pacific and land surface temperatures in Ethiopia with annual malaria risk in Oromia, based on confirmed cases of malaria between 1982 and 2005 [20].

### Enhancing National Climate Services (ENACTS)

The availability of high-quality climate data (both temperature and rainfall) suitable for national and local analyses has been challenging in Africa due to policy, resourcing, and technical constraints. As a result, researchers often use globally available climate products derived from satellite data or model outputs that lack the detailed and more accurate information provided by locally available observations from meteorological stations.

The Ethiopian health community has long expressed the need for quality-assured and locally relevant climate information as part of their infectious disease surveillance activities [33]. The ENACTS initiative emerged in Ethiopia in direct response to these expressed needs [34]. The initiative seeks to improve climate data availability, access, and use by national decision-makers. It combines rigorously evaluated station data (temperature, rainfall) from the NMA historical archive and globally available satellite and climate model reanalysis products [35, 36]. The ENACTS gridded climate data are disseminated by the NMA on request or via ‘Maprooms’ created using IRI Data Library software [37], and housed at NMA’s website. This unprecedented capability enables national meteorological agencies to provide quality-assessed and spatially and temporally complete climate information and associated services which out-compete traditional global products in terms of quality. This is because the latter normally accesses only a small fraction of the national observations provided through the global telecommunications system (GTS) of the World Meteorological Agency [38]. Similar ENACTS initiatives are being undertaken in over a dozen countries, including Rwanda [39].

The high spatial and temporal resolution of the ENACTS monitoring and historical data (4–5 km and aggregated at daily, decadal (10-day) or monthly time steps) starting from 1981, can be extracted for any grid point, or a vector file (such as an administrative boundary or river catchment) using the online Maproom facilities. The historical database can also be used to explore the relationship of external drivers, such as ENSO, to historical rainfall and temperature (both minimum and maximum) variations at any location and for any season. As ENACTS provides operational climate products and services, the system continues to evolve in response to user demands. While NMA maintains the ENACTS database and distributes the ENACTS temperature and rainfall data products following specific requests—it makes many derived products freely available online in the form of maps and graphs that can be manipulated by the user.

### Malaria epidemics and climate in Ethiopia

Malaria control (and elimination) in Ethiopia has long been a public health and national development priority [40]. Although four of the human malaria parasites have been reported from Ethiopia, the dominant species are *Plasmodium falciparum* and *Plasmodium vivax*, each accounting for about 60% and 40% of cases, respectively [41]. *Plasmodium vivax* is known to tolerate cooler climates than *P. falciparum* and has been found to be the dominant species in some highland regions [42].

Approximately 50% of the estimated 115 million Ethiopians live in cool highland areas, where malaria is restricted by low temperatures and numerous studies have shown that malaria in these areas is highly sensitive to climate anomalies [41–44], and trends [32].

Highland areas above 1500 m, mainly the escarpments of the Rift Valley and the western, central, and eastern areas, are especially prone to periodic epidemics [32, 42]. Highland-fringe areas with low transmission (between 1750 and 2000 m) are considered as highest in epidemic risk while occasional epidemics occur above 2000 m. The well-documented 1958; and 2002 and 2003 epidemics occurred in towns and villages as high as 2400 m and 2500 m [45, 46] both associated with unusually wet and warm years and following extensive drought periods. In neighbouring Eritrea, rainfall is the dominant climate control on malaria risk and the highland regions of Eritrea also exhibit vulnerability to malaria epidemics [47].

Since there is normally a low risk of infection in these highland areas, functional immunity in the population is low. Consequently, under the right conditions, severe epidemics with high-case fatality rates can occur that affect both adults and children.

The encroachment of endemic malaria to highland and highland-fringe areas beyond its traditional upper limit is now well documented in Ethiopia. For instance, the occurrence of endemic malaria above 2000 m was recorded between 1997 and 2010, and non-epidemic malaria was reported as high as 2200 m in highland areas of Ethiopia [48, 49]. This was assumed to be associated with the general warming of the climate that has been observed across eastern Africa [50], including Ethiopia [23] in recent decades.

### Non-climatic drivers of malaria epidemics in Ethiopia

Non-climatic drivers of epidemics are also known to aggravate malaria incidence and in areas where epidemics are also facilitated by climate. For example, epidemics of malaria were associated with the massive population movements that occurred during the 1980s and 1990s. These movements, from the drought-stricken highlands to the fertile malaria endemic lowlands, were associated with government resettlement and development programs and as well as military deployment [51]. Malaria epidemics that occurred during the mid-1990s also coincided with the decentralization of the vertical malaria control program and its integration with basic health services [52]. Workforce attrition, poorly aligned succession plans, weakened surveillance and limited vector control efforts in some regions undermined the malaria response during this transition period [53].

During the 2002 and 2003 large scale epidemic, a nationwide survey by the Ministry of Health detected treatment failure that surpassed the World Health Organization (WHO) recommended tolerable levels [54]. The same study demonstrated the significance of replacement of sulfadoxine/pyrimethamine and the introduction of artemether-lumefantrine as first-line drug for the treatment of falciparum malaria. A two-year drought, and consequent, low anti-malaria immunity alongside high levels of malnutrition likely paved the way for the 2002 and 2003 epidemic. It was a shocking epidemic in which WHO estimated up to 15 million of the then 65 million population were affected: a three-fold increase from the normal caseload [46].

Thus, ENSO events may impact malaria occurrence either through direct climatic influences (enhanced rainfall or higher temperatures promoting vector and parasite development and malaria transmission) or through indirect impacts on population vulnerability (e.g., through reduced immunity associated with drought-induced famine).

### Aim and objectives of this study

The aim of this study was to undertake exploratory analyses that can inform the use of climate information in Ethiopian malaria control and adaptation programmes. Thus, to achieve this aim three sets of analyses identified. Namely:

1. Explore the impact of the ENSO phases on the seasonal and spatial patterns of rainfall and temperature (minimum and maximum) across Ethiopia using ENACTS gridded climate data.
2. Review the occurrence of malaria epidemics (local and widespread) across Ethiopia as recorded in peer review and grey literature and explore their association with ENSO phases and climate variables – including drought.
3. Explore the relationship of observed rainfall, minimum and maximum temperature in the Amhara region with sea surface temperatures (ENSO, IOD and Tropical Atlantic) and their relationships to local and widespread malaria epidemics in Amhara region.
4. Provide recommendations on further development of climate informed malaria control and health adaptation activities including epidemic early warning systems.

## Methods

**Fig. 1:**
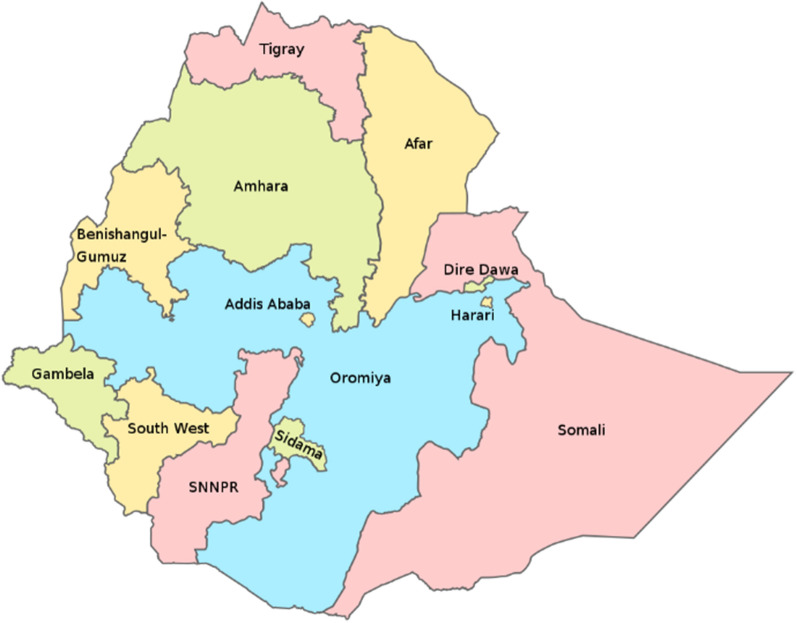
Regions of Ethiopia

The data and methods used to undertake the first three objectives of this study are set out below. A map of Ethiopia indicating the different regions is presented in Fig. 1. Note that two regions including Sidama and South West Ethiopia People’s Region were, at the time of the epidemic review, incorporated into SNNPR.

### Epidemic data

A detailed search of peer reviewed publications and ‘grey’ literature (reports) was made using Web of Science/Google search using the terms “Ethiopia” and “malaria” and “epidemic” or “outbreak” and year (and tracing of referenced articles therein) to identify records of malaria epidemics in Ethiopia for the period 1950–2014. Articles were identified that explicitly mentioned a single malaria epidemic/outbreak or set of epidemics that occurred anywhere in Ethiopia, within particular region(s) (e.g., the highlands), and/or within specific administrative boundaries i.e., region, zone or woreda/district. Notably, the terms of epidemic and outbreak were often used interchangeably. Language indicating the extent of the epidemic, for example, “local” or “widespread” were also noted. Where available, information was obtained on the temporal evolution of the epidemic (when it started, the month(s) of peak transmission season, and its end). If the author(s) simply stated an epidemic year or set of years without onset and/or end months, it was assumed that the epidemic started at any time between January and December of that year.

In this analysis, epidemics were defined by the authors of the original report without discrimination between the methods, indicators, thresholds, and/or mortality rates used by authors to distinguish an epidemic or an outbreak (see Additional file 1).

### Amhara epidemic data

Since the greatest number of reported epidemics in the literature were from Amhara, in part because of its intensive efforts to develop malaria epidemic early warning [55], this region was chosen for a more detailed analysis.

For Amhara a malaria index was created with years with no (0), localized (1) or widespread (2) epidemics. While there were many malaria epidemics throughout various parts of Ethiopia from the 1950s to the 1980s, earlier references to epidemics generally lacked monthly granularity. While the ENACTS climate data was available from 1981, the first epidemic that was widely documented with location specificity in Amhara took place in 1991. Consequently, the Amhara regional analysis is limited to epidemic occurrence data from 1991 to 2014.

### ENSO index

The ENSO phase (El Niño, Neutral or La Niña) for each 3-month period between 1950 and 2014 is defined according to the traditional Oceanic Niño Index (ONI) [56]. This index is calculated using SST anomalies, based on a 1981–2010 normal, in the eastern central Pacific. A season is considered to be in an El Niño (or La Niña) if it is part of at least five consecutive overlapping 3-month long seasons where the ONI is above 0.45 °C (or below − 0.45 °C). The threshold is further broken down into weak (with a 0.5 to 0.9 SST anomaly), moderate (1.0–1.4), strong (1.5–1.9) and very strong (≥ 2.0) events. As of going to press the last weak El Niño event was in 2018 whereas the last very strong event was 2015/2016. Three consecutive years of a moderate La Nina occurred in 2020–2022, which has been associated with extreme drought in south-east Ethiopia. Note that ENSO conditions invariably span more than one calendar year.

### Sea surface temperature data

Four SST indices were used in this study:

1. JAS NINO 3.4 Index [geographic box 5S to 5N, 170 to 120 W].
2. MAM NINO 3.4 Index [geographic box 5S to 5N, 170 to 120 W].
3. JAS IOD [geographic boxes: 50-70E, 10S-10N and 90-110E, 10S to 0].
4. JAS Tropical Atlantic SST index [geographic box is 20W-20E, 5S to 5N].

All SST data used were taken from the ERSST version 5 product [57] and downloaded from the IRI data library. Drought years in association with El Niño years were identified from the reports in the literature [26] and from the NMA archives.

### Climate data

ENACTS historical data (4 km spatial resolution and aggregated monthly) from 1981 for maximum temperature (Tmax), minimum temperature (Tmin) and rainfall were made available to the authors by the NMA and were visualised online in the NMA Maproom (see below) where the data products can be readily manipulated, and the final results downloaded as images.

#### Amhara ENACTS climate data

ENACTS Tmax and Tmin and rainfall data were extracted for the Amhara region and the JAS season.

#### ENSO Maprooms

ENSO Maproom were created using the IRI Data Library[37]; a version of which is installed at the NMA. These Maprooms are a collection of dynamic maps and other figures that are used to assess the past, monitor the current, and anticipate future climate conditions. The maps and figures can be manipulated as they query the underlying ENACTS data (http://213.55.84.78:8082/Maproom/index.html). The ENSO Maprooms include the historical probability (given in percentile) of seasonal average monthly rainfall, Tmax and Tmin falling within the upper (wet/hot), middle (normal), or bottom (dry/cool) one-third ("tercile") of the historical distribution in the country given the ENSO phases (El Niño, Neutral, La Niña) during that same season. The ENSO phase season is fixed as three consecutive months. To be consistent with the ENSO season this study present climate analyses with a 3-month season (e.g., JAS). Additional analyses using a 4-month climate season for the NMA *Kiremt* season (JJAS) did not produce any major changes in the results observed.

1. Impact of ENSO on the seasonal and spatial patterns of rainfall and temperature (minimum and maximum) in Ethiopia
  A detailed analysis of the spatial and temporal impact of ENSO phases, defined using the Oceanic Niño Index (ONI), on rainfall and temperature was undertaken using high resolution climate data made available through the ENACTS initiative. The spatial impact of ENSO phases on rainfall, Tmax and Tmin (above normal, normal and below normal) during the July–August-September (JAS) and the October–November-December (OND) was calculated for the periods of 1981–2015 using the NMA Maprooms.
2. The history of malaria epidemics in Ethiopia and ENSO
  Information on the geographic location, extent, and timing of the onset, peak and offset of the epidemic were extracted, where possible, to match the epidemics with concurrent ENSO phase and climate conditions prior or during the epidemic period. When the temporal information about the beginning, peak and end of the transmission period of the malaria epidemic was provided by the author, the corresponding ENSO phase was determined. However, if the author only provided the year of the epidemic, the entire year was assigned an ENSO phase if there were at least seven, 3-month long seasons of El Niño, La Niña, or Neutral ONIs. If an epidemic periodic included both Neutral and another ENSO phase the period was ascribed to El Niño or La Niña. On occasion both El Niño and La Niña phases took place within one calendar year, with each phase lasting at least three consecutive overlapping 3-month long seasons, the year was designated a “Combined ENSO”. As post-drought epidemics of malaria are considered common [2], the relationship between El Niño (weak, moderate, strong, very strong), drought and widespread epidemics was also explored.
3. The relationship of climate and SSTs to malaria epidemics in Amhara region
  The relationships between the SST, Amhara climate indices and epidemic index data were explored through several metrics. To start with, it was observed that in the 24 years of the epidemic data, there were 10 years of widespread epidemic, 6 years of localized epidemic and 8 years of no epidemic. Two thresholds were then selected for each climate variable to divide the data into below normal, near normal and above normal category. The purpose was to create three categories for each variable which roughly matched the data distribution in the malaria epidemic index data.

For the NINO 3.4 index the thresholds correspond with El Niño, Neutral and La Niña phases (− 0.5 °C and 0.5 °C—relative to the 1991–2014 mean). The other SST anomaly indices (IOD and Tropical Atlantic) were also calculated in the same way and, as with the NINO 3.4 index the thresholds were clearly equidistant from the (1991–2014 mean). The Amhara climate data (rainfall, Tmin and Tmax) were divided into three categories of above normal, near normal and below normal conditions based on the actual data. The threshold values are shown in Table 1 or the rainfall and temperature data, the values selected indicate a z-score (which indicates how much a given value differs from the mean) of ± 0.7 and ± 0.5 standard deviations, respectively.

**Table 1:** Lower and upper threshold values for each climate variable used in the analyses

Climate Variables	Lower threshold	Upper threshold
JAS NINO 3.4 (^o^C)	− 0.5	0.5
MAM NINO 3.4 (^o^C)	− 0.5	0.5
JAS IOD (^o^C)	− 0.3	0.3
JAS Trop Atlantic (^o^C)	− 0.15	0.15
JAS Amhara rainfall (mm)	674	772
JAS Amhara Tmax (^o^C)	24.1	24.7
JAS Amhara Tmin (^o^C)	13	13.3

The potential predictability of the malaria index against the SST and climate categories was then explored with a skill score commonly used in climate prediction science for three-by-three contingency tables: the Gerrity Skill Score (GSS) [58]. The Gerrity Skill Score was developed for monitoring precipitation forecasts and for guiding forecast system development. It is designed to accommodate the difficult distribution of rainfall and is considered less sensitive to sampling uncertainty than other established skill scores [59]. In a forecast system weather is partitioned into dry, normal and heavy precipitation. In this analysis, malaria is substituted for weather and partitioned into no, local and widespread epidemics (Table 2).

**Table 2:** Gerrity skill score contingency table

Epidemic status/magnitude	No epidemic predicted	Localized epidemic predicted	Widespread epidemic predicted
No epidemic occurs	p_11_	p_12_	p_13_
Localized epidemic occurs	p_21_	p_22_	p_23_
Widespread epidemic occurs	p_31_	p_32_	p_33_

The p_ij_ values are the proportion of observations in each category. The values on the main diagonal (p_11_, p_22_, p_33_) indicate when the forecast category and the observed category match, whereas the values in the off-diagonal indicate a mismatch between forecast and observed category. For each proportion value in the contingency table, a weight, s_ij_, is ascribed.

The GSS is then given by:

1 $$GSS= \sum_{ij}{s}_{ij}{p}_{ij}$$

Weights for accurate forecasts (i = j) are positive. Weights for inaccurate forecasts (i = / = j) are negative. A perfect forecast has p_11_ + p_22_ + p_33_ = 1 and a GSS of 1. If a forecast has no skill at discerning the correct category (pure random guessing), the GSS = 0. In this case, the positive weighted sum of the diagonal elements (s_11_p_11_ + s_22_p_22_ + s_33_p_33_) is equal in magnitude to the negative weighted sum of the off-diagonal elements (s_12_p_12_ + s_13_p_13_ + s_21_p_21+_ s_21_p_21_ + s_31_p_31_ + s_32_p_32_). A forecast with negative skill (worse than random guessing) has a negative value. While larger positive GSS values indicate better skill at forecasting categorical data, any positive GSS value represents an improvement over random guessing.

The weights, s_ij_ are determined in the following manner. First the “a” coefficients are determined as follows.

2 $${a}_{i}=\frac{1-\sum_{r=1}^{i}{p}_{r}}{\sum_{r=1}^{i}{p}_{r}}$$

where p_r_ is the proportion of observations in the corresponding predictand category (no epidemic, localized epidemic or widespread epidemic) and i is the category index. In this study, p_1_ = 8/24 = 0.333, p_2_ = 6/24 = 0.25 and p_3_ = 10/24 = 0.4167. Consequently, the values for a_1_ and a_2_ are 2 and 0.714, respectively. In a 3 × 3 category design, a_3_ is always 0.

After these “a” coefficients are determined, the weights are given as follows. For the diagonal elements (where i = j), the weights are given by

3 $${s}_{ii}=\frac{1}{K-1}\left(\sum_{r=1}^{i-1}{{a}_{r}}^{-1}+\sum_{r=i}^{K-1}{a}_{r}\right)$$

where K is the total number of categories (in this case 3). The weights for the diagonal elements in this study is given as follows: s_11_ = 1.357, s_22_ = 0.607 and s_33_ = 0.95.

The weights for the off-diagonal elements for j > i are given by:

4 $${s}_{ij}={s}_{ji}=\frac{1}{K-1}\left(\sum_{r=1}^{i-1}{{a}_{r}}^{-1}-\left(j-i\right)+\sum_{r=j}^{K-1}{a}_{r}\right)$$

The weights for the off-diagonal elements in this study are given as follows: s_12_ = s_21_ = − 0.143, s_23_ = s_32_ = − 0.25 and s_13_ = s_31_ = − 1.

## Results

### The impact of ENSO on the seasonal and spatial patterns of rainfall and temperature (minimum and maximum) in Ethiopia

A series of maps were created that present the historical probability (given in percentile) of 3-month seasonal average of monthly rainfall falling within the upper (dry/wet), one-third (“tercile”) of the 1981–2016 distribution in Ethiopia given the occurrence of El Niño or La Niña during that same season. Note that 33% is the approximate value if all categories are considered of equal likelihood of occurrence. Exemplars of these maps created using the NMA Maprooms for the main rainy seasons JAS and OND are presented below including targeted views of Amhara region. The maps indicate that different regions of Ethiopia (Fig. 1) experience different impacts from ENSO.

**Fig. 2:**
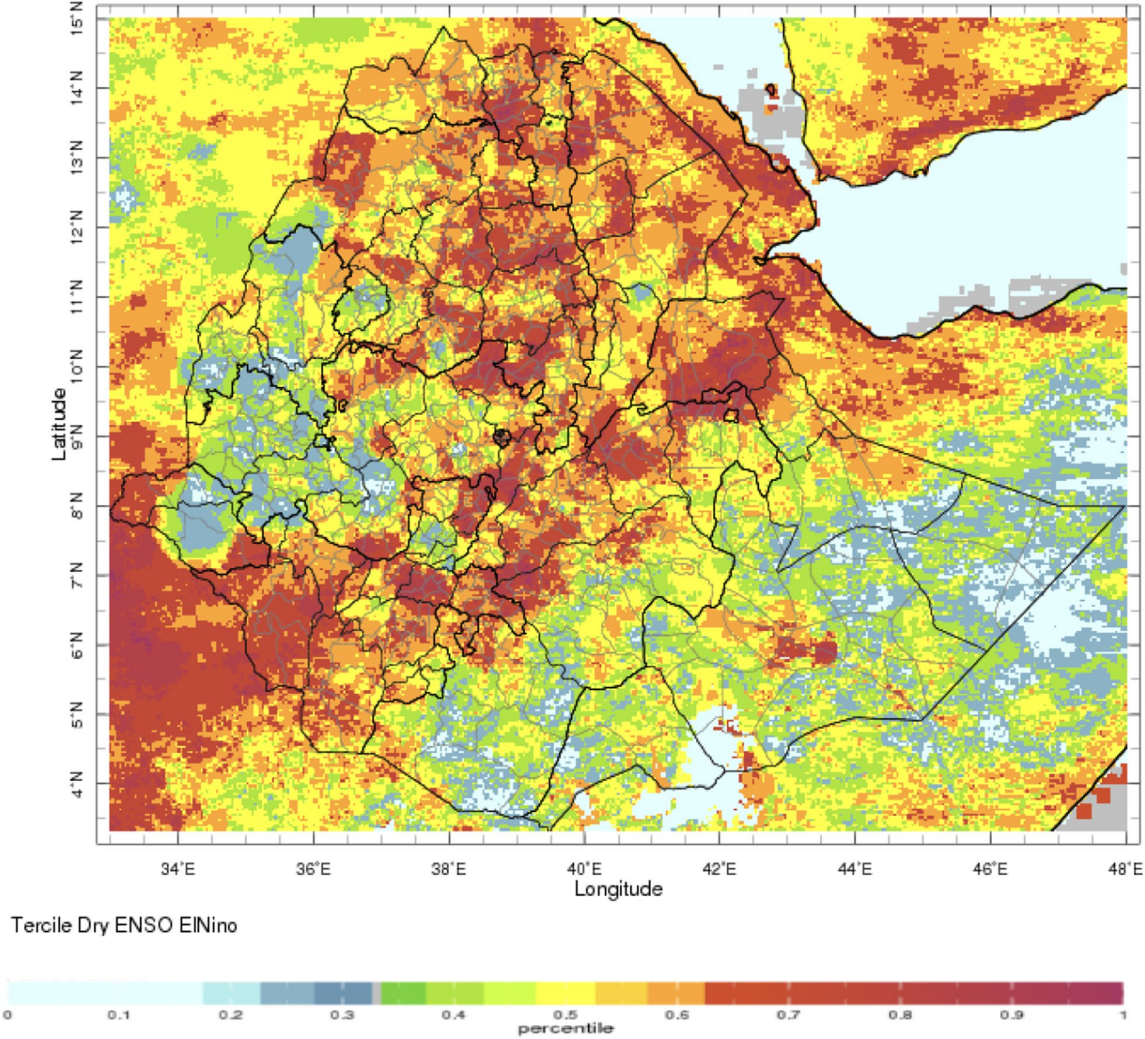
El Niño associated below normal rainfall impact for Jul–Sep (note the high probability of drought in the northern region with severe impact in the Amhara region—see below)—using monthly ENACTS from 1981 to 2016 (downloaded 10th August 2021)

**Fig. 3:**
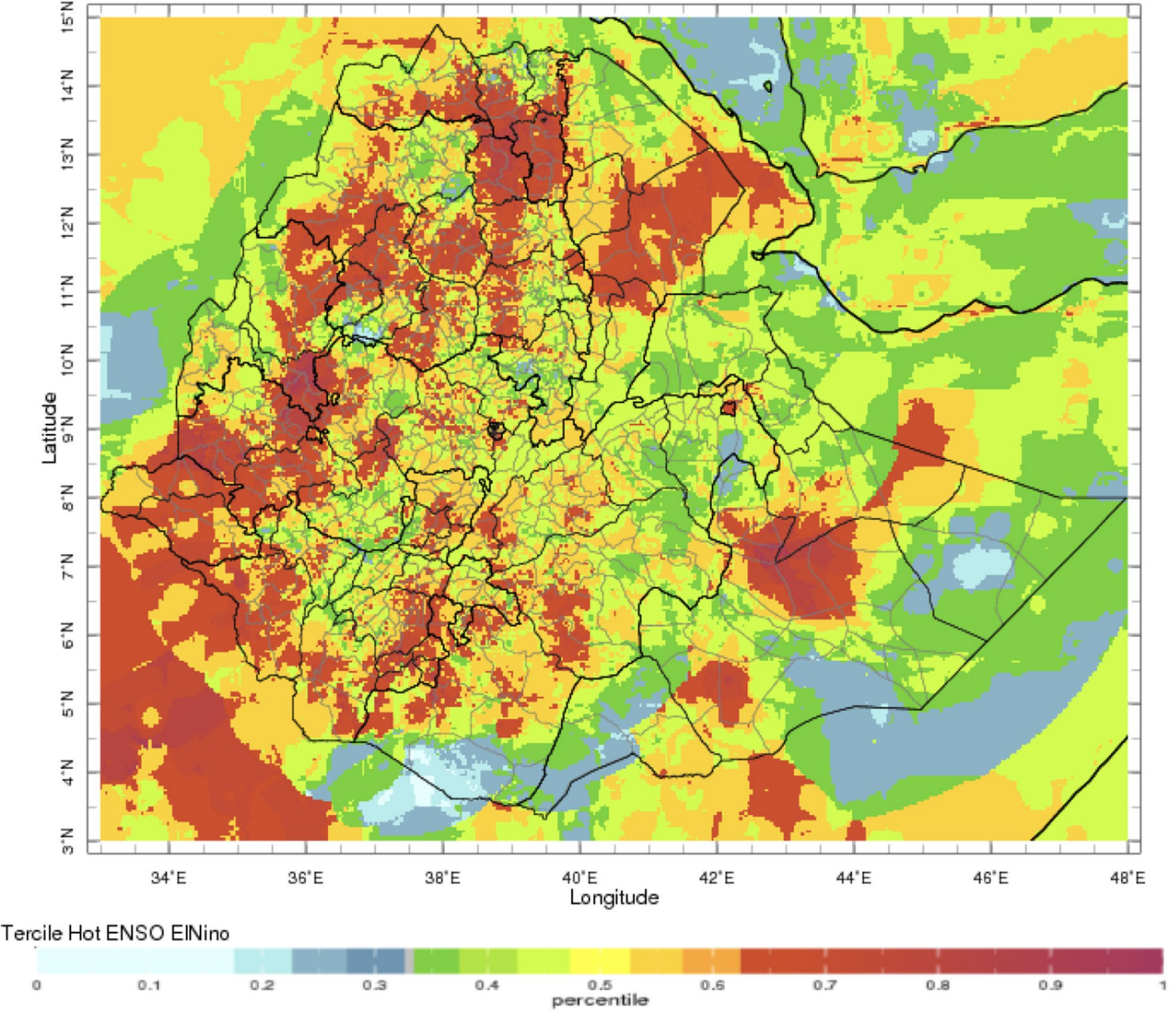
El Niño associated above normal maximum temperature impact for Jul–Sep (note that this continues across all seasons) while ENSO is current and often extends beyond the return to normal conditions—using monthly ENACTS from 1981–2016 downloaded 10th August 2021

**Fig. 4:**
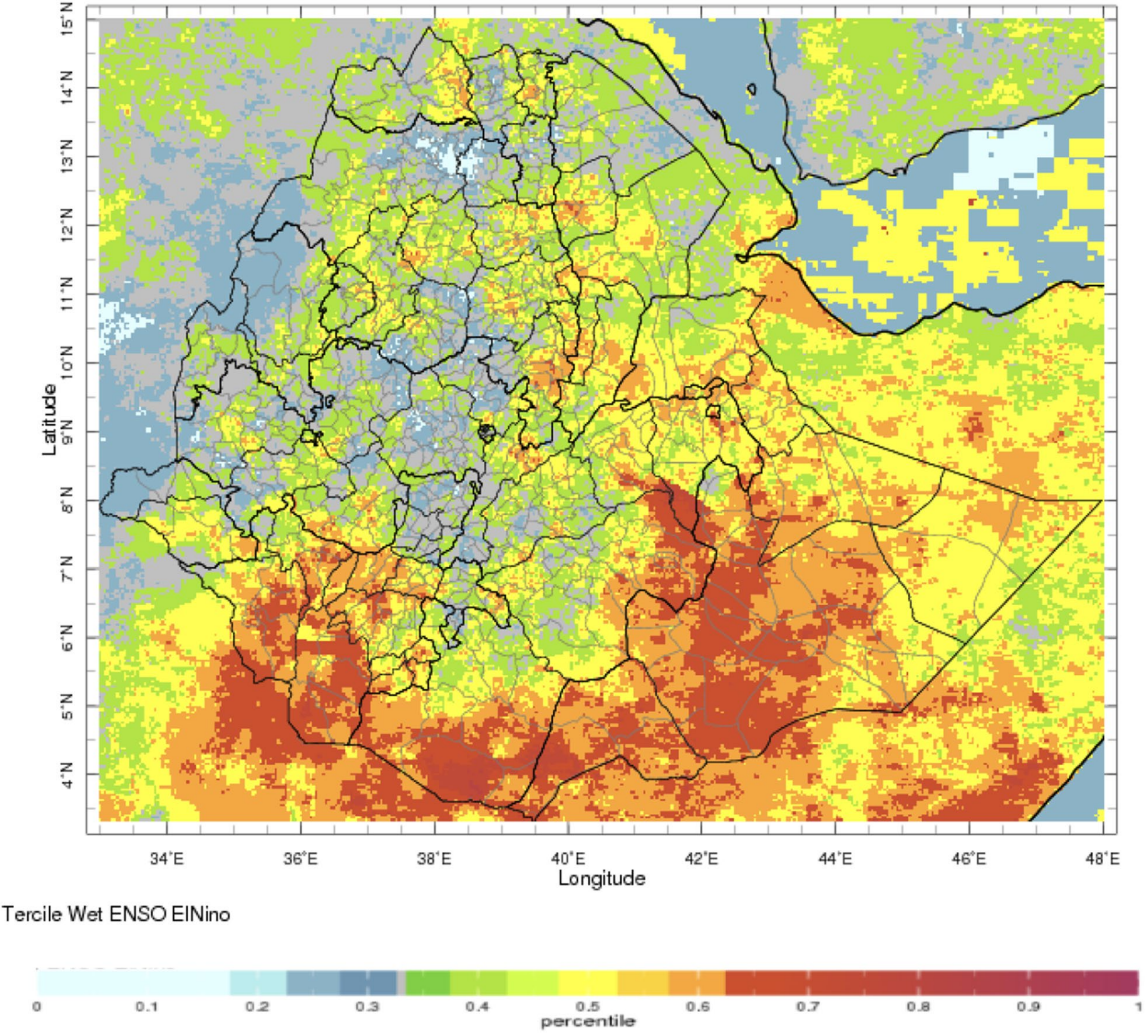
El Niño associated above normal rainfall for Oct–Dec (note the severe impact in the southern—eastern region of the country)—using monthly ENACTS from 1981 to 2016 downloaded 10th August 2021

**Fig. 5:**
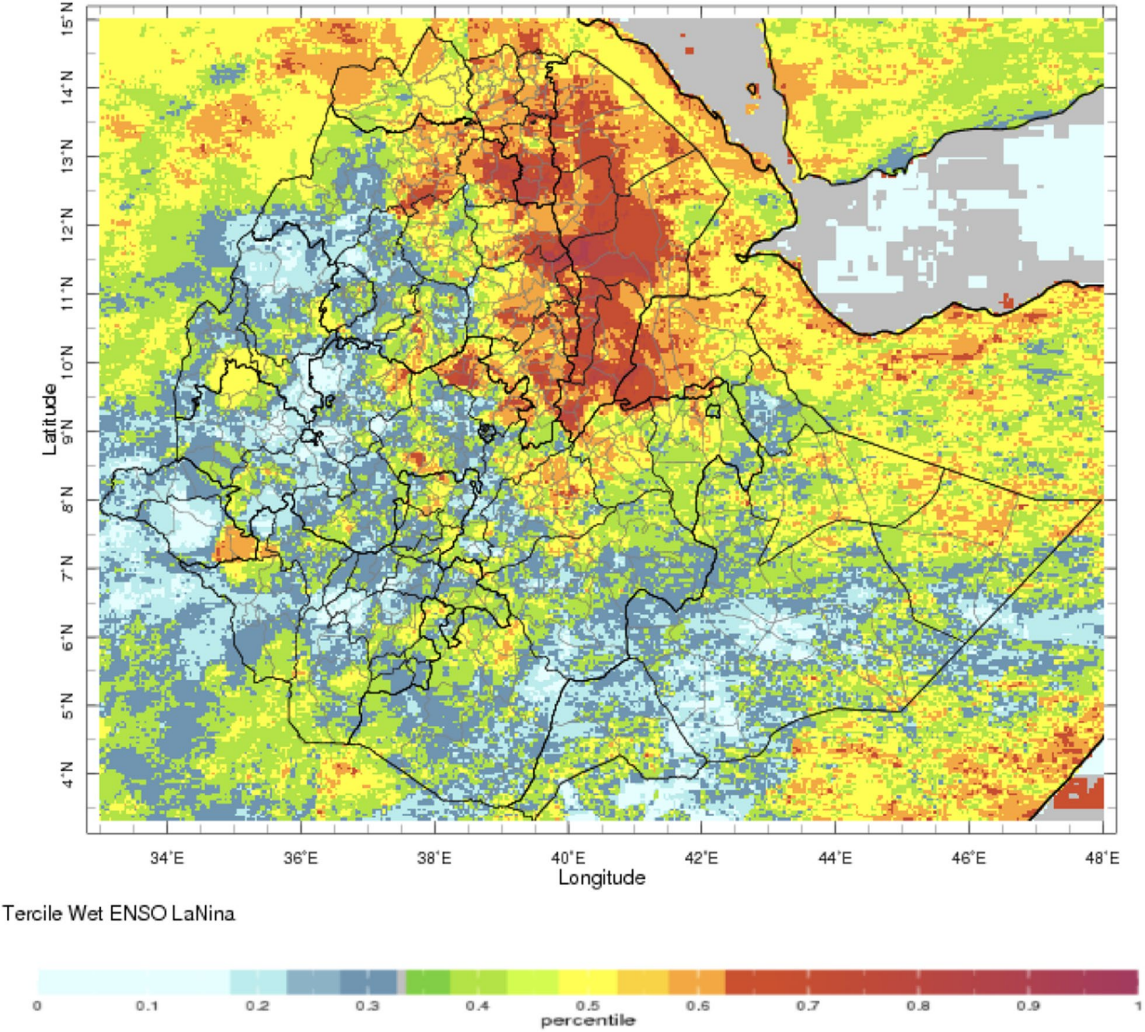
La Niña associated above normal rainfall for Jul–Sep (note the severe impact in the north-east—downloaded 10th August 2021

During the periods of analysis 1991–2014 for rainfall and temperature respectively, El Nino’s impact on rainfall and temperature is strongest over the north-western half of the country during the JAS season when the probability of low rainfall is high (Figs. 2, 3, respectively). In OND, El Niño years have a higher probability of high rainfall in south-east Ethiopia (Fig. 4). The occurrence of La Niña is associated with the approximately reverse effects (Fig. 5).

### The national history of malaria epidemics and ENSO

#### Epidemics identified and completeness of data records

Thirty-six epidemic malaria events in Ethiopia were identified from 31 publications and reports, during the period January 1950 to December 2014 (Additional file 1). The first reported epidemic was in 1953 and the last in 2012. Only 13 of the 36 events identified had complete information on the geographic location, extent and timing of the onset, peak and offset of the epidemic and could therefore be fully matched with the ENSO events. Epidemics with partial information were still used where possible in the analysis.

#### Epidemic extent

Of all reported, 19 epidemics were described or presented as “widespread”, and involved multiple woredas/ districts and or regions. Seventeen reports were described or presented as local epidemics, which were associated with one or more cities, woredas/ districts. Some reports described more than one epidemic.

#### Altitude

Epidemic events were in recorded highland regions (17/36) or in both highland and lowland regions (15/36). No publications described epidemics in lowland area alone. Three of the “widespread” events, and one “local” event did not record if the regions included highlands and/or lowlands.

#### ENSO and malaria epidemic occurrence

Of the 20 epidemics where the timing (in months) of the epidemic was available, 13 peaked during the Oct–Nov period following the JAS rainy season, one peaked in the April to June Season following the MAM rainy season and six had no record of the peak timing of cases.

14 malaria epidemic events (10 widespread) were found to be associated with El Niño; 12 (4 widespread) with La Niña; three (1 widespread) with Neutral years; and seven (4 widespread) with combined ENSO periods, characterized by a year in which SST modulated between both El Niño, La Niña, and neutral phases (Table 3).

**Table 3:**
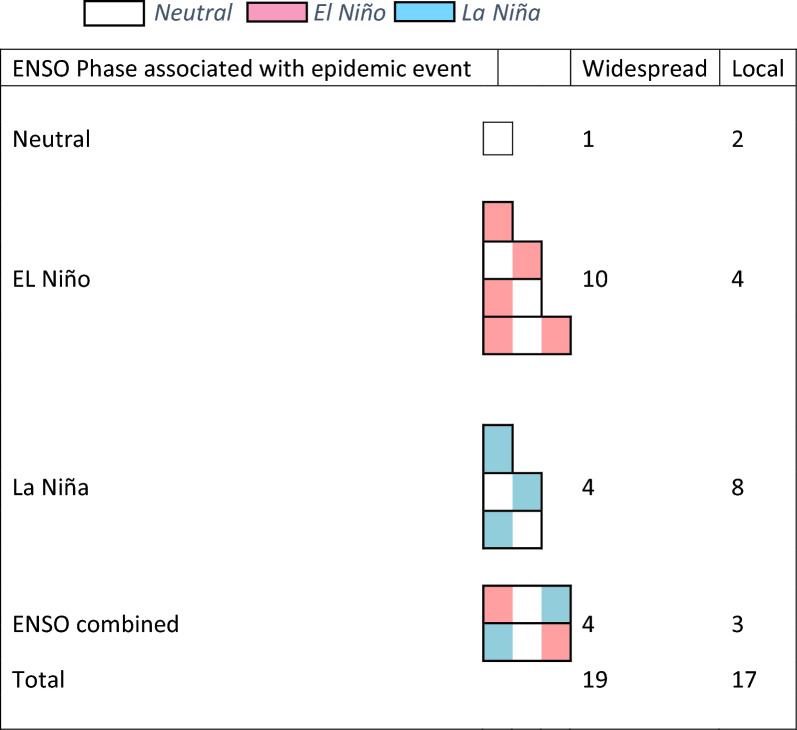
Malaria epidemic events associated with ENSO phases

The complexity of the ENSO and climate interactions along with malaria epidemic data limitations made it difficult to ascertain consistent relationships between these variables across the whole country. While widespread epidemics were predominantly associated with El Niño events, it was noted that epidemics occurred during and following all ENSO phases.

Of the 19 epidemic/outbreak events classified as widespread, 10 were associated with years classified as El Niño; two with La Niña; one with Neutral years; and two with ENSO periods (Table 3).

During the 65 years of the period under investigation (1950–2014) approximately half, (29 years: here calculated as running from July–June to align with ENSO events) were identified as having recorded widespread or local malaria epidemics/outbreaks, as defined by the original authors. Of these, 12 widespread epidemic events were associated with drought years. All strong or very strong El Niño events (eight) were associated with drought in Ethiopia and drought occurred in association with five of eight moderate El Niño events. Only two of the 11 weak El Niño events were associated with drought. Widespread epidemics occurred concurrent with El Niño years and following El Niño years with, or without, drought. Note that for these analyses the year 2015 was added as this was the most extreme ENSO event to occur and was associated with a very severe drought in Ethiopia [56]. No information on malaria following 2015 El Niño is presented (Table 4).

**Table 4:**
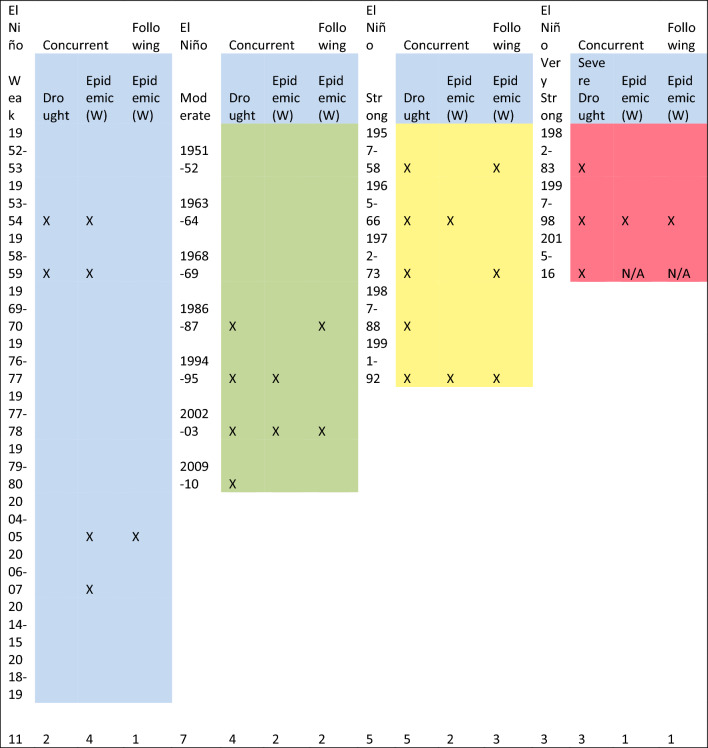
Association of drought and widespread epidemics concurrent and following El Niño Year

### The relationship of climate and SSTs to malaria epidemics in Amhara

#### Rainfall and temperature conditioned on ENSO

**Fig. 6:**
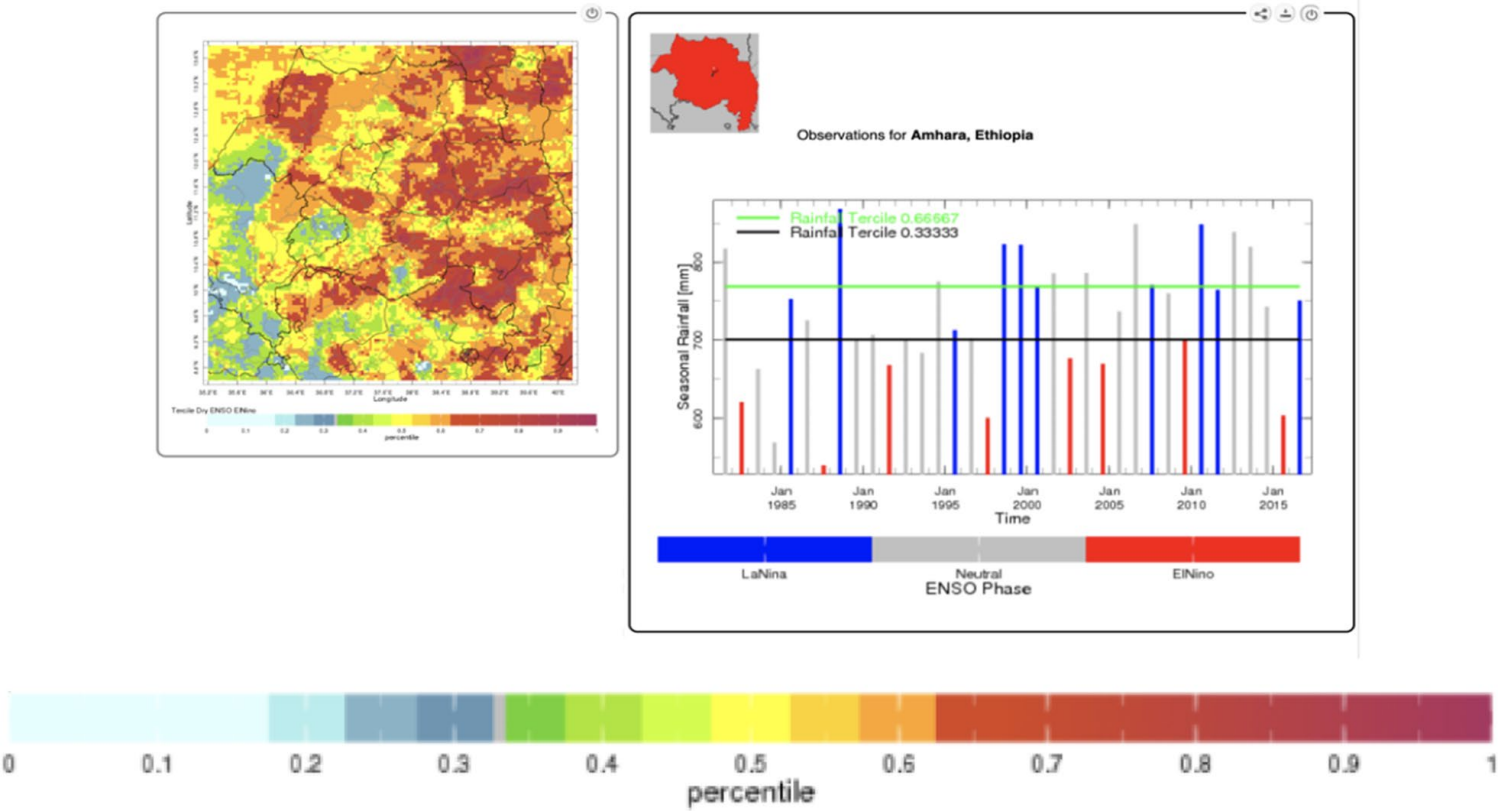
El Niño associated below normal rainfall for Jul–Sep for Amhara region only (1982–2016) with images and text taken directly from the NMA Maproom—downloaded 10th August 2021)

Seasonal rainfall amount for JAS conditioned on ENSO was visualised using the NMA Maproom website (Fig. 6). Note that unusually low rainfall is associated with El Niño (red column) with all rainfall amounts in the lowest tercile and 1987 and 1998 showing the most severe drought. Unusually high rainfall is associated with La Niña (blue column) with no rainfall amounts in the lowest tercile, 6/10 in the near normal tercile and 4/10 in the highest tercile.

**Fig. 7:**
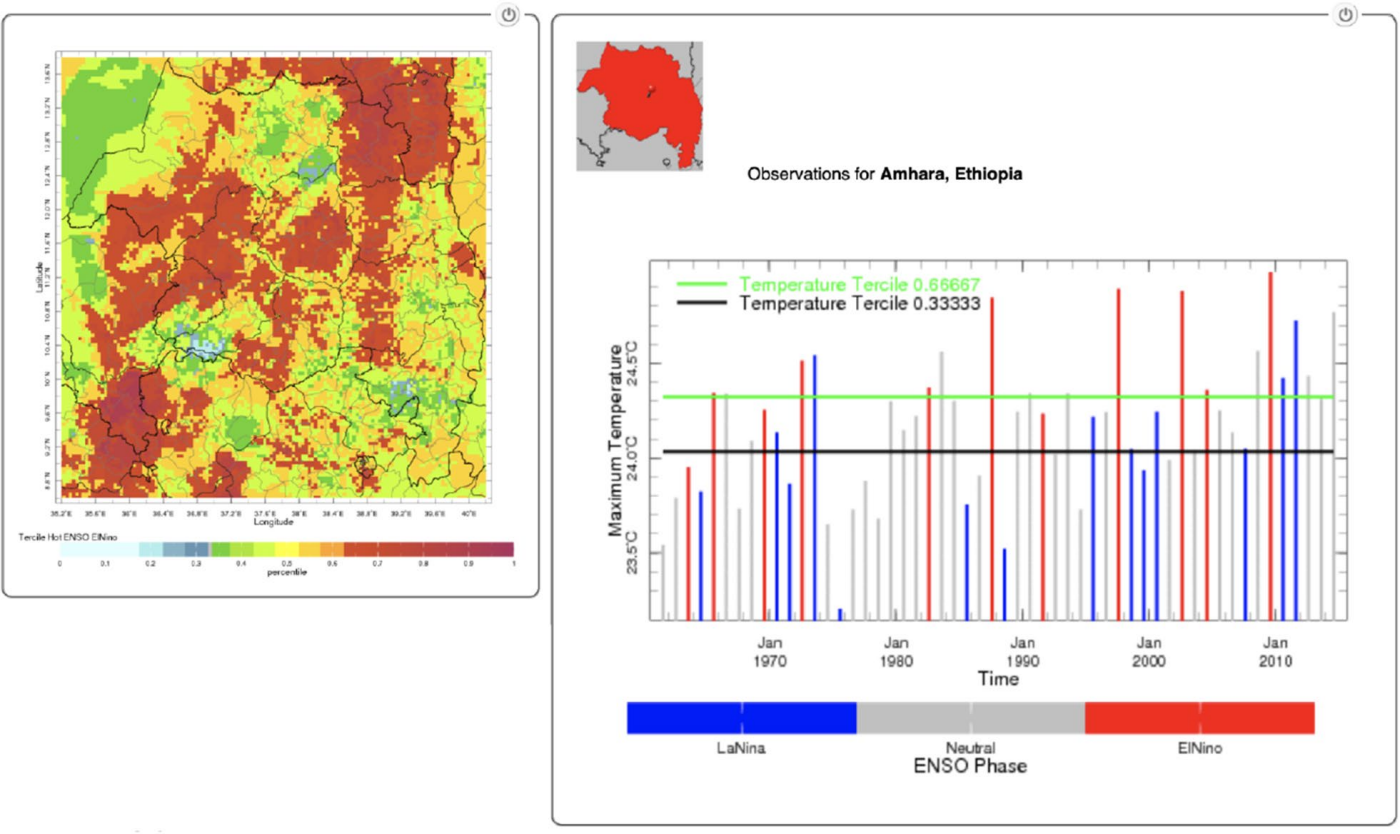
El Niño associated above normal maximum temperature for Jul–Sep (1961–2014) for Amhara region only with images and text taken directly from the NMA Maproom—downloaded 10th August 2021)

Seasonally averaged maximum temperature for JAS conditioned on ENSO was visualised using the NMA Maproom website (Fig. 7). The 4 warmest years were all associated with El Niño (red) whereas the four coolest years were associated with La Niña (blue).

Note the time series graphic indicates substantive warming over the time period of analyses that is consistent with decadal shifts and climate change trends [23].

#### Correlations between climate varibles.

The Pearson correlations between the climate variables for the 1991–2014 period described in Table 1 were tested and the results shown in Table 5. There is a strong negative correlation of (r = − 0.582, p < 0.05) between JAS NINO 3.4 Index and JAS Amhara rainfall (i.e., El Niño events are associated with drought in Amhara). Amhara JAS rainfall is also positively correlated with the Tropical Atlantic JAS SST anomaly (r = 0.383, p < 0.1). JAS Amhara maximum temperature and rainfall are negatively correlated (r = − 0.369, p < 0.1) and JAS Amhara minimum temperature is positively correlated with MAM NINO 3.4 (r = 0.382, p < 0.1).

**Table 5:** Correlations between the climate variables (based on the 1991–2014 time period)

Climate Variables	JAS NINO3.4	MAM NINO3.4	JAS IOD	JAS Tropical Atlantic	JAS Amhara rainfall	JAS Amhara Tmax	JAS Amhara Tmin
JAS NINO3.4	1	0.217	0.379*	− 0.362*	− 0.582**	0.287	0.124
MAM NINO3.4		1	− 0.358*	− 0.357*	− 0.229	− 0.222	0.382*
JAS IOD			1	0.167	0.029	0.041	− 0.189
JAS Tropical Atlantic				1	0.383*	0.039	0.260
JAS Amhara rainfall					1	− 0.369*	− 0.177
JAS Amhara Tmax						1	− 0.004
JAS Amhara Tmin							1

Note that given the length of record, the threshold correlation for 2-sided null hypothesis test p < 0.05 is ± 0.4 – values in this category are marked**. For p < 0.1 the threshold is ± 0.336 – values in this category are marked*

Bold text for the p<0.1 (*) numbers and bold underline text for the p<0.05 (**) number.

There are also significant (p < 0.1) correlations between several of the SST indices. These include correlations of -0.358 and -0.357 between the MAM NINO 3.4 and the JAS IOD and the JAS Tropical Atlantic indices, respectively. There is a positive correlation between the JAS NINO 3.4 SST anomaly and the JAS IOD index (r = 0.379, p < 0.1) and a negative correlation between the JAS NINO 3.4 SST anomaly and the JAS Tropical Atlantic Index (r = − 0.362, p < 0.1).

The significant correlations highlighted here should be considered in the context of a regional climatology that has complex dynamics, multiple time scales of global and regional climate variability and change, complex orography and multiple moisture sources for Ethiopia.

#### Trend analysis

The correlation of the various SST and climate indices with time was evaluated and while the SST indices did not show significant trends over time using either the 1981–2014 or 1991–2014 periods a longer time window may have provided a different result [60]. However, significant (p < 0.05) warming trends were observed, which are consistent with long-term trends that have been observed by others [23] and which are consistent with climate change predictions for the region [58, 61].

Wetting trends were identified in Amhara JAS rainfall. These statistically significant trends are shown in Table 6. However, in Eastern Africa care must be taken when attributing rainfall changes to climate change because of significant decadal variability observed during the MAM season [11].

**Table 6:** Based on a two sided Pearson correlation, statistically significant(p < 0.05) trends in rainfall and temperature in Amhara for two time periods

Time period	Amhara JAS rainfall	Amhara JAS Tmax	Amhara JAS Tmin
1981–2014	18 mm/decade	0.43 °C/decade	0.21 °C/decade
1991–2014	15 mm/decade	0.31 °C/decade	

#### Malaria index and ENSO phase in Amhara

Local and widespread epidemics were associated with every phase of ENSO in Amhara*.* For the four SST indices, Tmin and rainfall, a higher Gerrity score was obtained by assuming a positive correlation with the malaria index (i.e., warmer minimum temperatures or more rainfall were associated with widespread epidemics). For Tmax, the higher Gerrity score (0.287) was found assuming a negative correlation between the variables (i.e., cooler maximum temperatures were associated with widespread epidemics (Table 7). Note that cooler Tmax is usually associated with daytime cloud cover and possible rainfall.

**Table 7:** Average values of the SST and climate indices for the different malaria epidemic index categories (0–1–2) for Amhara and the associated Gerrity Skill Score

Climate indices	No epidemic (0)	Localized epidemic (1)	Widespread epidemic (2)	Gerrity skill Score
Temperature variables (^o^C)				
JAS NINO 3.4	0.094	− 0.159	0.085	0.079
MAM NINO 3.4	0.041	− 0.231	0.156	0.041
JAS IOD	− 0.165	0.025	0.031	− 0.012
JAS Trop Atlantic	− 0.111	0.231	0.160	0.187
JAS Amhara Tmax	24.6	24.5	24.2	0.287^a^
JAS Amhara Tmin	13	13.1	13.2	0.272
Rainfall variable (mm)				
JAS Amhara rainfall	689	767	724	0.249

^a^Note that the Gerrity Skill score for JAS Amhara Tmax is based on a negative correlation (i.e., cooler Tmax tend to be correlated with more widespread malaria epidemics). All other Gerrity scores shown are based on a positive correlation framework

For ENSO, the Gerrity skill score was 0.079 consistent with the observation of epidemics occurring in every ENSO phase. The value for IOD was even lower (0.012). The Gerrity Skill Score for JAS tropical Atlantic was much higher at 0.187.

While all Gerrity skill scores were below 0.3, the positive values provide an indication that, for Amhara, JAS season, high Tropical Atlantic SSTs, Tmin, and rainfall and low Tmax are weakly associated with localized and widespread malaria epidemics. Further exploration with longer time series of better-quality malaria data might prove helpful and a multi-variate analysis might be appropriate.

## Discussion

This exploratory analysis was undertaken using authoritative, publicly available, information on ENSO phases (and other SST climate drivers), high-resolution climate data from the NMA's ENACTS database and Maprooms and information on recorded malaria epidemics from varied peer review and 'grey' literature sources. The latter data source has considerable weaknesses in that malaria epidemic events are not consistently defined, and the specificity of information provided varied considerably between reports. Bias in reporting may also significantly influence the results. Epidemics may well have taken place that were not reported in the literature so “absence of an epidemic” cannot be construed as no epidemic took place. However, despite these weaknesses it is likely that all the major widespread epidemics are captured and some important lessons can be learned that are of relevance to Ethiopia’s national malaria control and elimination strategy [40] as well as the integration of health into the National Adaptation Plans. The ENACTS data for Ethiopia, created and disseminated by the NMA is a step change in the quality of climate data available for national decision-making. In summary:

The NMA Maprooms provide powerful visual insights for health users on the spatial and temporal variations in Tmin, Tmax and rainfall in Ethiopia and their interaction with ENSO phases. These Maprooms can make complex climatic relationships at the local, regional and national scales visible. The spatial and seasonal relationship between ENSO (and other oceanic drivers), rainfall and temperature across Ethiopia vary by climate variable, season, and region. Epidemics of malaria usually follow the June to September *Kiremt* rainfall season and occur predominantly in high altitude regions. There are no records of epidemics occurring solely in low lying areas or following OND rainfall (see Additional file 1).

Widespread epidemics may occur in any ENSO phase but were most commonly related with El Niño events, which are associated with higher maximum temperatures across Ethiopia and drought in the north-west region during the *Kiremt* rainy season. Few widespread epidemics followed La Niña events, which are often associated with higher rainfall and lower maximum temperatures in the north-west and drought in the north-east. This suggests that warmer temperatures are more important than unusually heavy rainfall in driving epidemics in Ethiopia.

In Amhara region in north-west Ethiopia, there was a strong relationship between ENSO phases and JAS rainfall, Tmin and Tmax. However, the relationship of these and other climate drivers to malaria risk in Amhara was mixed. Higher than average JAS SSTs in the Tropical Atlantic, rainfall and Tmin along with lower than average Tmax were weakly associated with elevated malaria epidemic risk following the JAS rainy season. No relationship with ENSO phases was observed. Warming trends in Amhara were consistent with observed and predicted warming associated with climate change in Ethiopia and the East African region. Positive wetting trends may be the result of decadal rainfall variability that has been observed in eastern Africa MAM season.

## Conclusion

This study reiterates the complexity of the Ethiopian climate and its relationship with ENSO phases and other oceanic drivers; predictability varying by climate variable, region, and season. The Maprooms and ENACTS data, created and disseminated by the NMA, provides an important resource for climate analysis by the health community. Local and widespread epidemics may occur in any ENSO phase but there are strong indications that El Niño years pose an increased risk for malaria epidemics in the September-December season in central, western, and northern highlands of Ethiopia following the *Kiremt* main rainy season (JAS). Higher temperatures, and rainfall are important factors in driving epidemics at a local and regional level.

The cyclical nature of El Niño (2–7 years) means that a global event is likely in the coming years following an extended La Niña period. Malaria control managers should be alert to any warnings of an emerging El Niño event. In addition, climate change is expected to continue its warming trends while increasing rainfall extremes (including extreme droughts) [62].

The deteriorating operation of health systems following the recent political crisis in the north of the country and elsewhere, along with projected warming trends and fluctuation of rainfall should alert malaria control managers to the likely climate risks to achieving malaria elimination by the end of this decade. Climate variability and change is also important for other infectious diseases, such as arboviral, water-borne and food borne-diseases along with non-communicable health outcomes such as malnutrition and heat stress. Significant improvements in the availability, access and use of climate information in Ethiopia over the last decade [63] can play an important role in informing current control programmes while preparing health sector responses to climate change through the national adaptation plans [3].

Based on the above the recommendations are that:

- NMA updates the publicly available Maprooms
- NMA adds the Tropical Atlantic Maprooms to their suite of available online services
- NMA provides colour palette choices in the Maproom.
- Future work concentrates on epidemic risk in the north, western and central highlands.
- Future analyses are undertaken at regional and sub-regional (woreda) and local level.
- Continuous improves in the quality and access of malaria data are prioritized
- The health and climate community in Ethiopia continue to work together to advance a practical climate adaptation agenda that is based on the best available understanding of climate and health issues across the country.

## Supplementary Information

**Additional file 1.** Historical malaria epidemics in Ethiopia from 1950 to 2015 from peer-reviewed and gray literature including ENSO conditions, altitude, extent, timing, duration, peak timing, and climate information.

## Data Availability

Malaria epidemic data generated or analysed during this study are included in this published article [Additional files]. The climate data that support the findings of this study are available from; Link for ERSSTv5 data (via IRIDL): https://iridl.ldeo.columbia.edu/SOURCES/.NOAA/.NCDC/.ERSST/.version5/index.html?Set-Language=en. Link for NMA maproom page: http://213.55.84.78:8082/maproom/index.html. Link for ENACTS data page through IRIDL (extracting the data will require permission of Ethiopian NMA): http://iridl.ldeo.columbia.edu/SOURCES/.Ethiopia/.NMA/.ENACTS_v7/.ALL/. Maproom maps and graphs are publicly available. The ENACTS data are the property of Ethiopia's NMA. Data are however available upon reasonable request with permission of NMA.

## Abbreviations

ENACTS: Enhancing National Climate Services
ENSO: El Niño Southern Oscillation
GSS: Gerrity Skill Score
IOD: Indian Ocean Dipole
JAS: July–August–September
MAM: March–April–May
NINO 3.4: Nino 3.4 SST Index
NMA: National Meteorological Agency
NMME: North American Multi-Model Ensemble
OND: October–November–December
ONI: Oceanic Niño Index
SST: Sea surface temperatures
Tmax: Maximum temperature
Tmin: Minimum temperature
WHO: World Health Organization
