# Supplementary material for: El Niño and other climatic drivers of epidemic malaria in Ethiopia: new tools for national health adaptation plans
Source: Malar J. 2023 Jun 24;22:195. doi: 10.1186/s12936-023-04621-3 (PMC10290321; doi:10.1186/s12936-023-04621-3)
Supplement: Supplementary file 1 — Additional file 1. Historical malaria epidemics in Ethiopia from 1950 to 2015 from peer-reviewed and gray literature including ENSO conditions, altitude, extent, timing, duration, peak timing, and climate information. [file 12936_2023_4621_MOESM1_ESM.docx]

| **No. and associated references** | **Dominant ENSO conditions** | **Altitude** | **Widespread/**  **Local** | **Extent of Epidemic** | **Month/ Year** | **Duration of Epidemic** | **Peak Cases** | **Above Normal Min Temp in Lit** | **Above Normal Temp (ONLY) in Lit** | **Above Normal Rainfall in Lit** |
| --- | --- | --- | --- | --- | --- | --- | --- | --- | --- | --- |
| 1^[1]^ | El Niño | **H** | W | Gondar area/Dembia Plain (near Lake Tana) (**North Amhara**), Kobbo-Chercher Plain (**North East Amhara**), Awash Valley (**Oromia - Northeast of Addis Ababa - Central**), Jimma area (**West Oromia**), the Rift Valley lakes area (**Central Oromia**), Baco area (**West Oromia**) | Year | 1953 | Oct-Nov 1953 | N/A | N/A | N/A |
| 2^[2]^ | El Niño | **H** | W | across the **Northern and Central Highlands** - Bahir Dar (Amhara), Lake Tana region as well as most highland areas of Shoa, Gojam, Wollo and various parts of Wollega (Oromia), Arussi, Harar, Sidamo (SNNPR) and Tigray | Mon | June - Dec 1958 | Oct 1958 | Yes^5^ | Yes^2^ | Yes^2^ |
| 3^[3]^ | ENSO | **H** | **L** | Gondar (**North Amhara**) | Year | 1964 | N/A | N/A | N/A | N/A |
| 4^[4]^ | ENSO | **L & H** | W | considered similar to to 1958 epidemic in scope but not intensity | Year | 1965 | N/A | N/A | N/A | N/A |
| 5^4,^^[5]^ | ENSO | **L & H** | W | including Debre Zeit/Bishoftu Sector (**Central Oromia**) | Year | (1) 1973, (2) 1973 | (1) N/A, (2) Oct 1984 | Yes^5^ | N/A | N/A |
| 6^5^ | El Niño | **H** | L | Debre Zeit/Bishoftu Sector (**Central Oromia**) | Year | 1980 | Nov 1980 | N/A | N/A | N/A |
| 7^4^ | La Niña | **L & H** | L | Parts of Ethiopia, including Debre Zeit/Bishoftu Sector (**Central Oromia**) Central Oromia - similar in scope to 1958 but less intense - considered similar in scope to the 1958 epidemic but less intense | Year | (1) 1981-82, (2) 1981 | (1) N/A, (2) July 1981 | N/A | N/A | N/A |
| 8^4^ | El Niño | N/A | L | Parts of Ethiopia including central Oromia | **Year** | 1981-82 | N/A | N/A | N/A | N/A |
| 9^5^ | La Niña | **H** | L | Debre Zeit/Bishoftu Sector (**Central Oromia**) | **Year** | 1985 | June 1985 | N/A | N/A | N/A |
| 10^[6]^ | El Niño | **H** | L | Shire Province (North Tigray Region) | **Year** | 1986 | N/A | Yes^5^ | N/A | N/A |
| 11^6^ | El Niño | **H** | L | Shire Province (North Tigray Region) | **Year** | 1987 | N/A | Yes^5^ | N/A | N/A |
| 12^5,^^[7],^^[8]^ | La Niña | **L & H** | W | including Central Oromia, Oromia Highlands - West and Central Oromia including Debre Zeit/Bishoftu Sector (Central Oromia), and Oromia Highlands | Mon | (1) 1988, (2) 1988, (3) April-Nov 1988 | (1) N/A, (2) Oct-Nov 1988, (3) N/A | Yes^5^ | N/A | N/A |
| 13^[9]^ | Neutral | **H** | L | Northern Highlands (includes Tigray) | **Year** | 1990 | N/A | N/A | N/A | N/A |
| 14^6^ | El Niño | **L & H** | **W** | North Tigray, West Amhara, Central Oromia including Shire Province (North Tigray), around Lake Tana (West Amhara), and in highland areas of Shewa (Central Oromia), south and southwest of Addis Ababa (Central Oromia) | **Month** | June-Oct 1991 | N/A | Yes^5^ | N/A | N/A |
| 15^5,8,^^[10]^ | El Niño | **L & H** | W | including Debre Zeit/Bishoftu Sector (Central Oromia), Oromia Region and Rift Valley (SNNPR) | Year | (1) 1992, (2) 1991-92, (3) 1992 | (1) Oct-Nov 1992, (2) N/A, (3) N/A | Yes^5^ | N/A | N/A |
| 16^[11]^^,^^[12]^ | Neutral | **L & H** | W | including SNNPR | Month | Feb-Jun 1994 (SNNPR) | (1) N/A, (2) N/A | N/A | Yes^12^ | N/A |
| 17^11,12^ | El Niño | **L & H** | W | including Wollo region/Dessie (Amhara); Shiraro region (Tigray); the districts of Zwai (Lake Ziway), Butajira, Alaba, Nazret (Adama), and Metahara; Lake Tana area; Jimma Town (Oromia), as well as Amhara regions and central Rift Valley | Month | (1) Sept-Dec 1994, (2) Sept-Nov 1994 (Tigray and Amhara regions and central Rift Valley) | (1) N/A, (2) N/A | N/A | Yes^12^ | N/A |
| 18^11,^^[13]^ | El Niño | **L & H** | L | Bahirdar and Jimma Towns (Oromia), | Month | (1) Mar-Apr 1995, (2) Mar-Apr 1995 (Bahirdar and Jimma Towns); | (1) N/A; (2) N/A | N/A | Yes^12^ | N/A |
| 19^11,13^ | La Niña | **L & H** | W | Wollo region/Dessie (Amhara); Shiraro region (Tigray); the districts of Zwai (Lake Ziway), Butajira, Alaba, Nazret (Adama), and Metahara; Lake Tana area; Jimma Town (Oromia), as well as Amhara and SNNPR regions | Month | (1) Mar-Apr 1995, (2) Mar-Apr 1995 (Bahirdar and Jimma Towns); Jun-Sept 1995 (Oromia, Amhara and SNNPR regions) | (1) N/A; (2) N/A | N/A | Yes^12^ | N/A |
| 20^8^ | La Niña | **L & H** | L | Oromia Region | Year | 1995-96 | N/A | N/A | N/A | N/A |
| 21^8,^^[14],^^[15],^^[16]^ | El Niño | **L & H** | W | Akaki Town district (Oromia), and Adolana Wadera (Adola), Hagere Mariam, Odo Shakiso, and Galan Abaya Districts (Oromia Region) | Month | (1) Jul 1997-Dec 1999, (2) 1997-99 | (1) Oct-Dec 1997, (2) N/A | Yes^14^ | N/A | N/A |
| 22^8,14,15,16^ | El Niño | **L & H** | W | Akaki Town district (Oromia), (2) Adolana Wadera (Adola), Hagere Mariam, Odo Shakiso, and Galan Abaya Districts (Oromia Region) | Month | (1) Jul 1997-Dec 1999, (2) 1997-99 | (1) Oct-Dec 1997, (2) N/A | Yes^14^ | N/A | N/A |
| 23^8,14,15,16^ | La Niña | **L & H** | W | Akaki Town district (Oromia), and Western Harerge and West Shoa (Shewa) Zones (Oromia), (3) Debre Zeit Sector (Oromia), (4) Burie and Waktola (Amhara) | Month | (1) Jul 1997-Dec 1999, (2) Jul-Dec 1998, (3) 1997-98, (4) May 1998-Jan 1999 | (1) N/A, (2) N/A, (3) Oct-Dec 1998, (4) Aug-Sept 1999 | Yes^14^ | N/A | N/A |
| 24^8,14,15,16^ | La Niña | **L & H** | W | Akaki Town district (Oromia), (2) Western Harerge and West Shoa (Shewa) Zones (Oromia), (3) Debre Zeit Sector (Oromia), (4) Burie and Waktola (Amhara) | Month | (1) Jul 1997-Dec 1999, (2) Jul-Dec 1998, (3) 1997-98, (4) May 1998-Jan 1999 | (1) N/A, (2) N/A, (3) Oct-Dec 1998, (4) Aug-Sept 1999 | Yes^14^ | N/A | N/A |
| 25^8,^ ^[17]^ | La Niña | **H** | L | Bako Tibe/Baco (Oromia), (2) Adolana Wadera (Adola), Hagere Mariam, Odo Shakiso, and Galan Abaya Districts (Oromia) | Month | (1) 2000, (2) June-Aug 2000 | (1) N/A, (2) N/A | N/A | Yes^8^ | Yes^8^ |
| 26^[18]^ | La Niña | **H** | L | Amhara - including Akasha Guagusa, Artuma Fursi, Berehet, Dawa Chefa, Fagita Lemoka, Guba Lafto, Libokemkem, Mecha, Merhabete, Minjar Shenkora, Sekota, Tacharmacho | Month | Sept-Dec 2002 | Oct-Nov 2001 |  |  |  |
| 27^[19],18^ | El Niño | **H** | **W** | across the Ethiopian Highlands (Amhara, Oromia, SNNPR, Tigray) and including Akasha Guagusa, Artuma Fursi, Berehet, Dawa Chefa, Fagita Lemoka, Guba Lafto, Libokemkem, Mecha, Merhabete, Minjar Shenkora, Sekota, Tacharmacho | Month | (1) 2002-03 (2) Sept-Dec 2002 | (1) N/A (2) Oct-Nov 2002 | Yes^19^ | N/A | N/A |
| 28^18,19^ | Neutral | **H** | **W** | across the Ethiopian Highlands (Amhara, Oromia, SNNPR, Tigray and including - Akasha Guagusa, Artuma Fursi, Berehet, Dawa Chefa, Fagita Lemoka, Guba Lafto, Libokemkem, Mecha, Merhabete, Minjar Shenkora, Sekota, Tacharmacho | Month | (1) Mar-Dec 2003 (2) Sept-Dec 2003 | (1) Oct-Dec 2003 (2) Oct-Nov 2003 | Yes^19^ | N/A | N/A |
| 29^7,18^ | El Niño |  | **W** | Akasha Guagusa, Artuma Fursi, Berehet, Dawa Chefa, Fagita Lemoka, Guba Lafto, Libokemkem, Mecha, Merhabete, Minjar Shenkora, Sekota, Tacharmacho | Month | (1) 2004-05 (2) Sept-Dec 2004 | (1) N/A (2) Oct-Nov 2004 | N/A | N/A | N/A |
| 30^7,18^ | ENSO |  | **W** | across parts of Ethiopia including - Akasha Guagusa, Artuma Fursi, Berehet, Dawa Chefa, Fagita Lemoka, Guba Lafto, Libokemkem, Mecha, Merhabete, Minjar Shenkora, Sekota, Tacharmacho | Year | (1) 2006-07 (2) Sept-Dec 2014 | (1) N/A (2) Oct-Nov 2014 | N/A | N/A | N/A |
| 3^17,18^ |  | N/A | **W** | across parts of Ethiopia including Akasha Guagusa, Artuma Fursi, Berehet, Dawa Chefa, Fagita Lemoka, Guba Lafto, Libokemkem, Mecha, Merhabete, Minjar Shenkora, Sekota, Tacharmacho | Month | (1) 2006-07 (2) Sept-Dec 2014 | (1) N/A (2) Oct-Nov 2014 | N/A | N/A | N/A |
| 32^18^ | La Niña | **H** | **L** | Akasha Guagusa, Artuma Fursi, Berehet, Dawa Chefa, Fagita Lemoka, Guba Lafto, Libokemkem, Mecha, Merhabete, Minjar Shenkora, Sekota, Tacharmacho | Month | Sept-Dec 2007 | Oct-Nov 2007 |  |  |  |
| 33^18^ | La Niña | **H** | **L** | Akasha Guagusa, Artuma Fursi, Berehet, Dawa Chefa, Fagita Lemoka, Guba Lafto, Libokemkem, Mecha, Merhabete, Minjar Shenkora, Sekota, Tacharmacho | Month | Sept-Dec 2008 | Oct-Nov 2008 |  |  |  |
| 34^8,^^[20],^^[21],^^[22],1^ | ENSO | **H** | **L** | North and South Gondar (Amhara), (2) Jimma Town (Oromia), (3) Mareko and Meskana districts (SNNPR), (4) Local - Akasha Guagusa, Artuma Fursi, Berehet, Dawa Chefa, Fagita Lemoka, Guba Lafto, Libokemkem, Mecha, Merhabete, Minjar Shenkora, Sekota, Tacharmacho (Amhara) | Year | (1) Jan 2009-Dec 2010, (2) 2009-10, (3) 2009-10, (4) Sept-Dec 2009 | (1) Sept-Oct 2009, (2) Sept-Oct 2009, (3) Oct-Nov 2009, (4) Oct-Nov 2009 | Yes^21,22^ | N/A | Yes^22^ |
| 35^21,22^ | ENSO | **H** | **L** | North and South Gondar (Amhara), (2) Jimma Town (Oromia), (3) Mareko and Meskana districts (SNNPR), (4) Local - Akasha Guagusa, Artuma Fursi, Berehet, Dawa Chefa, Fagita Lemoka, Guba Lafto, Libokemkem, Mecha, Merhabete, Minjar Shenkora, Sekota, Tacharmacho (Amhara) | Year | (1) Jan 2009-Dec 2010, (2) 2009-10, (3) 2009-10, (4) Sept-Dec 2010 | Oct-Nov 2010 | Yes^21,22^ | N/A | Yes^22^ |
| 36^[23]^ | La Niña | **H** | **L** | Ankesha District (Amhara) - Azena Kebele, Sumatra Kebele, Ayehu Kebele | Month | Mar-Dec 2012 | Apr-Jun 2021 | N/A | N/A | N/A |

1. Covell, S. G. (1957). "Malaria in Ethiopia." J. Trop Medicine & Hyg **60**: 7-16.
2. Fontaine, R., Najjar A, Prince J. (1961). "The 1958 Malaria epidemic in Ethiopia." Am J Trop Med Hyg. **10**(795-803).
3. Mengistu M, Maru M and Ahmed Z. (1979) Malaria in Gondar, Ethiopia, 1975-1978: a review of 435 cases with special emphasis on cerebral malaria. Ethiop Med J 1979 Jul;17(3):57-62.
4. Gebremariam N., Abdulahi Y., Mebrate A. 1988. Malaria, pp. 136–150. In Zein Z. A., Kloos H. (eds.), Ecology and disease in Ethiopia. Ministry of Health, Addis Ababa, Ethiopia.
5. Tulu, A. (1996). Determinants of malaria transmission in the highlands of Ethiopia: the impact of global warming on morbidity and mortality ascribed to malaria. PhD. London.
6. Teklehaimanot, A. (1991). Report of a follow-up mission to Ethiopia 27 October - 2 November 1991. Malaria epidemics in Ethiopia with particular reference to the war-affected northern regions. . Geneva, WHO.WHO
7. Presidents Malaria Initiative (2008). Malaria Operational Plan (MOP) Ethiopia FY 2008.
8. Deressa W, Olana D, Chibsa S. (2005). Community participation in malaria epidemic control in highland areas of southern Oromia, Ethiopia. Ethiopian Journal of Health & Development 2005 (19). 1. 3-10
9. Ghebreyesus, T. A., Z. Tadese, D. Jima, E. Bekele, A. Mihretie, Y. Y. Yihdego, T. Dinku, S. J. Connor and D. P. Rogers (2008). "Public health and weather services–climate information for the health sector." Bulletin of the World Meteorological Organisation **57**(4): 256–261.
10. World Health Organization 1997. World malaria situation in 1994, Weekly Epidemiological Record, 72: 269-276.
11. Anon. The present malaria situation in the Amhara Region. Bahir Dar: Amhara Regional Health Bureau; 1997.
12. Woime E. Changes in the spread of Malaria in Ethiopia: Case study from Awassa and Hossana area 2006-2007. Norway: Telemark University College; 2008.
13. National Malaria Control Team, E. P. H. I., World Health Organization, Addis Ababa University and the INFORM Project (2014). "An epidemiological profile of malaria in Ethiopia. A report prepared for the Federal Ministry of Health, Ethiopia, the Roll Back Malaria Partnership and the Department for International Development, UK
14. Woyessa, A, Gebre-Michael, T, Ali, A, & Kebede D, 2002 Malaria in Addis Ababa and its environs: assessment of magnitude and distribution. *The Ethiopian Journal of Health Development*, *16*(2).
15. Woyessa, A and Ali A. (2003). "Highland fringe malaria and challenges in its control: The lesson from Akaki Town." Ethiopian Medical Journal **41**(4): 293-300.
16. Woyessa, A., Gebre-Michael T, Ali, A. (2004). "An indigenous malaria transmission in the outskirts of Addis Ababa, Akaki Town and its environs." Ethiop J Health Dev. **18**(1): 2-7.
17. Kiszewski, A. E. and A. Teklehaimanot (2004). "A review of the clinical and epidemiologic burdens of epidemic malaria." American Journal of Tropical Medicine and Hygiene **71**(2_suppl): 128-135. <http://www.ajtmh.org/cgi/content/abstract/71/2_suppl/128>
18. Wimberly, M.C., Midekisa, A., Semuniguse, P., Teka, H., Henebry, G.M., Chuang, T.-W. and Senay, G.B. (2012), Spatial synchrony of malaria outbreaks in a highland region of Ethiopia. Tropical Medicine & International Health, 17: 1192-1201. <https://doi.org/10.1111/j.1365-3156.2012.03058.x>
19. Negash K, Kebede A, Medhin A, Argaw D, Babaniyi O, Guintran JO, Delacollette C. Malaria epidemics in the highlands of Ethiopia. East Afr Med J. 2005 Apr;82(4):186-92. doi: 10.4314/eamj.v82i4.9279. PMID: 16122086.
20. Alemu, A., G. Abebe, W. Tsegaye and L. Golassa (2011). "Climatic variables and malaria transmission dynamics in Jimma town, South West Ethiopia." Parasites & Vectors **4**: 30.
21. Alemu, K., A. Worku and Y. Berhane (2013). "Malaria Infection Has Spatial, Temporal, and Spatiotemporal Heterogeneity in Unstable Malaria Transmission Areas in Northwest Ethiopia." PLOS One 10.1371/journal.pone.0079966. 10.1371/journal.pone.0079966
22. Woyessa, A., Deressa W, Ali A, Lidtjorn B (2012). "Prevalence of malaria infection in Butajira area, south-central Ethiopia." Malar J **11**(84).
23. Lake, M.W. Mebratu, M, Mehari, D, & Dessie., K. Epidemiological Analysis of Malaria Outbreak in Ankesha District, Awi Zone, Amhara Region, Ethiopia, 2012: Weaknesses in Control Measures and Risk Factors. *Science Journal of Public Health.* Vol. 4, No. 2, 2016, pp. 132-137.
